# Blockade of PD-1 and LAG-3 expression on CD8+ T cells promotes the tumoricidal effects of CD8+ T cells

**DOI:** 10.3389/fimmu.2023.1265255

**Published:** 2023-09-28

**Authors:** Jiajia Ma, Shufang Yan, Ying Zhao, Huifang Yan, Qian Zhang, Xinxia Li

**Affiliations:** ^1^ Department of Pathology, Xinjiang Medical University Affiliated Tumor Hospital, Urumqi, Xinjiang, China; ^2^ Department of Critical Care, Medicine of Karamay Central Hospital, Karamay, Xinjiang, China; ^3^ Department of General Practice, Third People’s Hospital of Xinjiang Uygur Autonomous Region, Urumuqi, Xinjiang, China

**Keywords:** diffuse large B-cell lymphoma, tumor microenvironment, immune checkpoint inhibitors, tumor immune escape, prognosis

## Abstract

**Background:**

The diffuse large B-cell lymphoma (DLBCL) has the highest incidence of all lymphomas worldwide. To investigate the functions of lymphocyte activation gene 3 (LAG-3) and programmed cell death 1 (PD-1) in tissues and peripheral blood of patients with DLBCL, the expression of LAG-3 and PD-1 genes in DLBCL-TCGA were analyzed.

**Methods:**

*LAG-3* and *PD-1* mRNA levels in DLBCL were analyzed using data from The Cancer Genome Atlas (TCGA) database. Utilize the Genotype-Tissue Expression (GTEx) database for assessing the variance in the expression of LAG-3, PD-1, and other associated factors between the tissues of DLBCL patients and healthy individuals. Immunohistochemistry was applied to detect the expression of LAG-3 and PD-1 levels in 137 cases of DLBCL tissues and 20 cases of reactive lymphoid hyperplasia. The prognostic value of LAG-3 and PD-1 were assessed using the Kaplan-Meier curve. The Estimation of Stromal and Immune cells in Malignant Tumor tissues using Expression data (ESTIMATE) and ssGSEA algorithm were used to explore the immune microenvironment of DLBCL. Additionally, the expression and co-expression of LAG-3 and PD-1 were detected on CD4 and CD8 T cells in peripheral blood samples from 100 cases of DLBCL tissues and 30 cases of healthy individuals using flow cytometry.

**Results:**

According to TCGA database, *LAG-3* and *PD-1* gene expression levels were significantly up-regulated in DLBCL tissues. LAG-3 and PD-1 levels were also strongly positively correlated with those of most infiltrating immune cells. Overall survival of patients with high *LAG-3* and *PD-1* co-expression was significantly shorter than that of patients with low co-expression. In DLBCL patients, LAG-3 and PD-1 were highly expressed in peripheral blood CD8^+^ T cells. In addition, LAG-3 was highly expressed in CD4^+^ T cells, while the expression of PD-1 in CD4^+^ T cells of DLBCL patients showed no significant difference compared to healthy individuals. Additionally, CD8^+^ T cells and SU-DHL6/OCI-LY3 from patients with DLBCL were co-cultured *in vitro*; after addition of LAG-3 and/or PD-1 inhibitors alone, an increased perforin and granzyme B secretion levels by CD8^+^ T cells were detected, as well as an increase in the overall proportion of tumor cells undergoing apoptosis.

**Conclusion:**

High LAG-3 and PD-1 levels significantly inhibit CD8^+^ T cell function, resulting in weakened ability to kill tumor cells. Combined LAG-3 and PD-1 blockade can restore CD8^+^ T cell function and provides a potential avenue for development of personalized cellular immunotherapy for DLBCL.

## 1 Introduction

The prevalence of diffuse large B-cell lymphoma (DLBCL) is the highest among all lymphomas globally, making up 40% of all non-Hodgkin lymphomas (1, 2). R-CHOP, the standard first-line treatment option, is effective for more than 60% of patients (3, 4). However, approximately 30% of patients do not respond to chemotherapy or develop relapsed/refractory DLBCL, with poor treatment outcomes (5–7), leading to an increased mortality rate (8). Therefore, the search for more effective treatments for DLBCL remains a top priority.

In recent years, immune checkpoint inhibitors (ICIs) have become a focus of intense research in tumor immunotherapy, which has achieved remarkable therapeutic results in solid tumors (9, 10). Programmed cell death protein 1 (PD-1) is expressed on the surface of antigen-stimulated T cells (11), and PD-1 upregulation causes T cell receptor signaling attenuation, leading to inhibition of T cell activation, proliferation, and cytokine production (12). As antigens are cleared, PD-1 expression decreases rapidly. However, persistent presence of antigens causes ongoing PD-1 expression, eventually resulting in T-cell depletion (13).

Preliminary research has indicated that PD-1 inhibitors are effective in treating hematologic malignancies, such as classical Hodgkin’s lymphoma and follicular lymphoma, and may also be useful in DLBCL (14). However, the objective response rates were only 28% and 21% for patients with melanoma and kidney cancer receiving PD-1 treatment, respectively (15). Overall, PD-1 inhibitors have demonstrated remarkable efficacy in treating several malignancies, but their effects in DLBCL are unclear (16). Furthermore, previous studies on the prognostic impacts of PD-1^+^ tumor infiltrating lymphocytes (TILs) in lymphoma have reached contradictory conclusions (14, 17). The makeup of the tumor microenvironment is determined by interactions between immune cells and cytokines, which can limit the ability to mount an anti-tumor immune response. These interactions are not constant, and there are differences in the abilities of various lymphocyte types to recognize tumor cells. Hence, the prognostic impact of TILs on tumors is uncertain.

Accordingly, it is necessary to investigation of other immune checkpoints and identification of less toxic and resistant immunotherapy options. Combining immunosuppressive therapies could be a solution for overcoming resistance to PD-1 inhibitors. Among the new-generation of ICIs, lymphocyte activation gene 3 (LAG-3) serves as a crucial target for the development of tumor immunotherapies (18, 19). LAG-3 comprises 498 amino acids (20, 21). During chronic infections and tumor stimulation of the immune system, T cells exhibit elevated LAG-3 expression on their surface and their proliferation and cytokine production are decreased, ultimately resulting in T cell failure (22). Collaboration between LAG-3 and PD-1 promotes cancer cell immune evasion and obstructing both LAG-3 and PD-1 is considerably more effective and less harmful for treating solid tumors that are advanced or have metastasized (20, 22). There are few studies on LAG-3 expression in DLBCL, and further research to determine whether it can be an effective therapeutic target for DLBCL and improve PD-1 inhibitor resistance is warranted.

In this study, we founded that LAG-3 and PD-1 were widespread and abundant in peripheral blood from patients with DLBCL and correlated with prognosis and disease stage. Aberrant expression of immunosuppressive receptors on T lymphocytes is associated with impaired CD8^+^ T cell proliferation and differentiation, imbalance in subpopulation ratios, and abnormal cell function. Combined treatment with LAG-3 and PD-1 inhibitors restored CD8^+^ T cell secretion of cytokines and was more effective than either inhibitor alone.

## 2 Materials and methods

### 2.1 Data sources and preprocessing

Data from 47 DLBCL tumors in The Cancer Genome Atlas (TCGA-DLBCL) and 444 normal control (NC) tissues were downloaded from the University of California Santa Cruz (UCSC) database (https://genome.ucsc.edu). The 4th edition of the World Health Organization (WHO) Classification of Tumors of Hematopoietic and Lymphoid Tissues in 2018 was taken as the basis (23). Paraffin-embedded tissue specimens were collected from 137 patients with pre-chemotherapy DLBCL and 20 patients with reactive lymphoid hyperplasia in the First Affiliated Hospital of Xinjiang Medical University from 2011 to 2020 (**Supplementary Table 1**). Patients with pre-chemotherapy DLBCL diagnosed pathologically (n = 100) and 30 control samples from healthy peripheral blood samples at the Tumor Hospital affiliated with Xinjiang Medical University, collected from 2021 to 2022, were included (**Supplementary Table 2**).

### 2.2 Analysis of differentially expressed genes

The Linear fitting methods, Bayesian analysis and T-test algorithms to identify differentially expressed genes (DEGs) between DLBCL and NC by “limma” package (24) in R software. A total of 2136 differential genes were identified, of which 1313 were up-regulated and 823 down-regulated according to |log2 foldchange|>1 and *P* < 0.05.

### 2.3 Gene functional enrichment analyses

Gene Ontology (GO), including biological processes, cellular components (CCs), and molecular functions (MFs), and Kyoto Encyclopedia of Genes and Genomes (KEGG) analyses were applied to explore the function of DEGs using the “clusterProfiler” package (25) in R software.

### 2.4 Analysis of levels of LAG3, PD-1, and related factors in DLBCL and controls

Levels of *LAG3*, *PDCD1*, and other related factors in TCGA and GTEx database were analyzed for differences and the T-test used to assess the significance of differential expression between cancer and normal tissues.

### 2.5 Analysis of immune cell infiltration

CIBERSORT (26), an immune tumor biological computational tool, was used to calculate immune cell levels and evaluate the infiltration status of each sample.

### 2.6 Immunohistochemistry

Tissue microarrays were immersed in citrate buffer and left to cool to room temperature, then treated with 3% hydrogen peroxide for 10 min, followed by addition of a drop goat serum after washing with PBS, and incubating at 37°C for 20 min. Next, samples were incubated with anti-PD-1 (Working fluid, Catalog Number: UMAB199, ZSGB-BIO) or anti-LAG-3 (1:2000, Catalog Number: 16616-1-AP, Proteintech) and chromogenic immunoassays performed with DAB after washing in PBS, followed by hematoxylin re-staining. Human tonsils served as positive control specimens for both antibodies. Negative control specimens were DLBCL tissue treated with phosphate buffer in place of antibody. Final scores were obtained by averaging frequency scores from two replicate tests conducted for each DLBCL case.

PD-1 positivity was localized to the cell membrane or cytoplasm of TILs, with median = 4 (range, 0–54) PD-1^+^ TILs per high power field (HPF); ≤ 4; PD-1^+^ TILs/HPF was considered low expression and > 4 PD-1^+^ TILs/HPF as high expression. LAG-3 positivity was also localized to the cell membrane or cytoplasm of TILs (median = 11, range = 0–103 LAG-3^+^ TILs/HPF); ≤ 11 LAG-3^+^ TILs/HPF was considered low expression, with > 11 considered high expression.

### 2.7 Cell culture

The DLBCL cells (OCI-LY3 and SU-DHL-6) were cultured in RPMI-1640 (Thermo Fisher Scientific, USA) supplemented with 10% fetal bovine serum and 1% penicillin and streptomycin at 37°C and 5% CO_2_. Anti-LAG-3 monoclonal antibody (0.5 nm/ml, MedChemExpress, USA) and/or anti-PD-1 monoclonal antibody (0.5 nm/ml, MedChemExpress, USA) were added into OCI-LY3 and SU-DHL-6 cells, respectively. Peripheral blood CD8^+^ T cells from patients with DLBCL were sorted using immunomagnetic beads (Becton, Dickinson and Company, USA) and co-cultured with OCI-LY3 or SU-DHL-6 cells.

### 2.8 Blocking assay

The CD8^+^ T cells obtained by MACS were placed in RPMI-1640 complete medium containing 10% FBS and culture overnight at 37°C, 5% CO_2_. At the same time, inoculate logarithmic growth SU-DHL6 and OCI-LY3 cells into a 24-well plate, with 6×10^4^ cells per well, and culture overnight. The next day, the already adhered SU-DHL6 and OCI-LY3 cells were incubated separately with anti-PD-1 or/and anti-LAG-3 monoclonal antibodies and isotype controls at room temperature for 2 h. After that, 4×10^5^ CD8^+^ T cells were added to each well and incubated at 37°C for 12 h. Finally, flow cytometry analysis was performed.

### 2.9 Flow cytometry

Freshly collected sodium heparin (100 μl) was added to each of two flow tubes. The two sets of fluorescently labeled antibodies were added to each of the two tubes: 1) CD4 and CD8 cells were labelled with 5 μl PD-1-PECy7, LAG3-PE, CD4-FITC, and CD8-PCP5.5; 2) CD4-FITC and CD8-PCP5.5 were used as negative controls. Cells were incubated for 15 min at room temperature in the dark, then 2 mL 1× erythrocyte lysate buffer was added per 100 μl of whole blood, and red blood cells lysed at room temperature for 8–12 min, until the cell suspension was transparent. Cells were then washed and centrifuged (3,000 g, 15 min), supernatants discarded, and 300 μl PBS added. CD4^+^ T cells were gated as T helper lymphocytes and CD8^+^ T cells as cytotoxic T lymphocytes. For detection of tumor cell apoptosis, after co-culture, CD8^+^ T cells were sorted and 5 μl Annexin V/PE (Becton, Dickinson and Company, USA) and 10 μl 7-AAD (Becton, Dickinson and Company, USA) added to the remaining cancer cells, mixed gently, and incubated. After incubation cells were analyzed by flow cytometry.

### 2.10 Statistical analysis

Data analysis and visualization were both conducted using Graphpad Prism 8.0 and Xiantao Academic database, which as a public information platform used for analyzing a large amount of sample data and visualizing the results (https://www.xiantaozi.com/). Pearson correlation analysis was applied to analyze the relationships of LAG-3 and PD-1 levels with immune cells. The Student’s T test was used for comparisons between two groups. *P* < 0.05 was considered a statistically significant difference.

## 3 Results

### 3.1 LAG-3 and PD-1 expression were up-regulated in DLBCL

**Figure 1:**
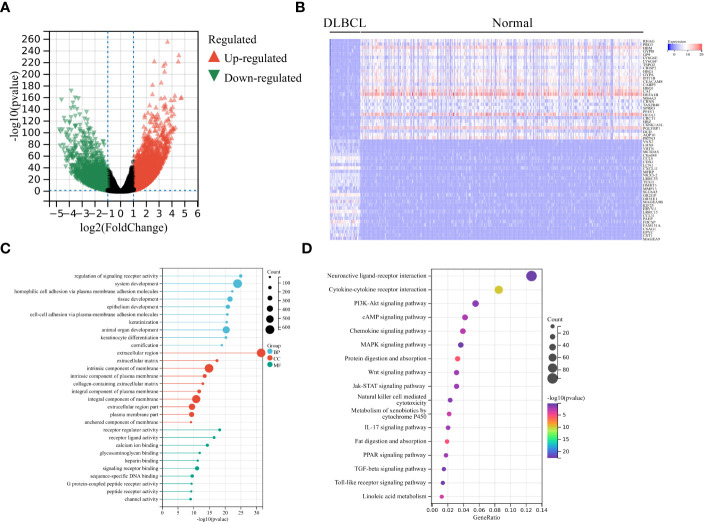
Analysis of differentially expressed genes (DEGs). **(A)** Volcano plot showing significant DEGs in DLBCL tumors in TCGA-DLBCL relative to normal tissues from GTEx. Significantly up-regulated and down-regulated genes are shaded in red and green, respectively. **(B)** Heatmap of the top 30 significantly increased (red) or decreased (blue) genes between TCGA-DLBCL and normal tissues. GO analysis **(C)** and KEGG pathway enrichment analysis **(D)** of significant DEGs.

**Figure 2:**
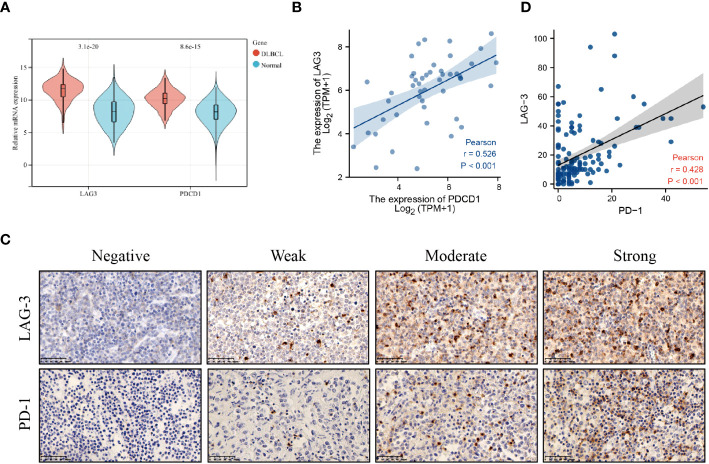
LAG-3 and PD-1 levels were increased in DLBCL. **(A)** LAG-3 and PD-1 levels in DLBCL and normal lymph nodes. **(B)** Correlation between LAG-3 and PD-1 levels in DLBCL tissues. **(C)** PD-1 and LAG-3 levels in tissues detected by immunohistochemistry. **(D)** Linear correlation analysis of LAG-3 and PD-1 levels in DLBCL tissues.

Firstly, we screened 2136 DEGs, including 1313 up-regulated genes and 823 down-regulated genes, from 48 DLBCL-TCGA and 444 normal tissues from GTEx (**Figures 1A, B**). Most DEGs mainly involved in the “regulation of signaling receptor activity”, “system development”, “PI3K-Akt signaling pathway” and “Wnt signaling pathway” (**Figures 1C, D**). The GO enrichment analysis showed that in DLBCL patients, changes in gene expression related to system development, organ development, tissue development, epithelial cell differentiation, cellular keratinization, extracellular processes, and cellular adhesion occurred, thereby affecting the originally normal physiological environment in DLBCL patients (**Figure 1C**). In addition, KEGG pathway analysis shows significant changes in genes involved in the PI3K-Akt, cAMP, MAPK, Wnt, JAK-STAT, TLR, IL-17, TGF-β, and chemokine-related signaling pathways in DLBCL patients, suggesting significant alterations in multiple signaling transduction pathways in DLBCL patient cells. It is worth noting that there have been significant changes in the genes involved in the cytokines-cytokine receptor interaction, as well as in cell cytotoxicity, in DLBCL patients, which suggests that immune function is disrupted in DLBCL patients (**Figure 1D**). The mRNA level of both LAG-3 and PD-1 was noticeably elevated in DLBCL (**Figure 2A**) and levels of LAG-3 and PD-1 were positively correlated with one another (**Figure 2B**).

PD-1 and LAG-3 were widely expressed and co-expressed on DLBCL TILs (PD-1^+^ TILs, n = 68, 49.6%; LAG-3^+^ TILs, n = 65, 47.4%; and PD-1^+^LAG-3^+^ TILs, n = 45, 32.8%) (**Figure 2C**; **Supplementary Tables 3**, **4**). The relationships among PD-1 and LAG-3 expression and clinical features of DLBCL patients were also analyzed. PD-1 expression was significantly associated with Eastern Cooperative Oncology Group (ECOG) score and Ann-Arbor stage, while LAG-3 levels were significantly associated with primary tumor site and lactate dehydrogenase (LDH) level. Further, co-expression of PD-1 and LAG-3 was significantly associated with Ann-Arbor stage and PD-1 levels were positively correlated with those of LAG-3 on TILs in DLBCL (**Figure 2D**).

### 3.2 LAG-3 as an independent risk factor for DLBCL

**Figure 3:**
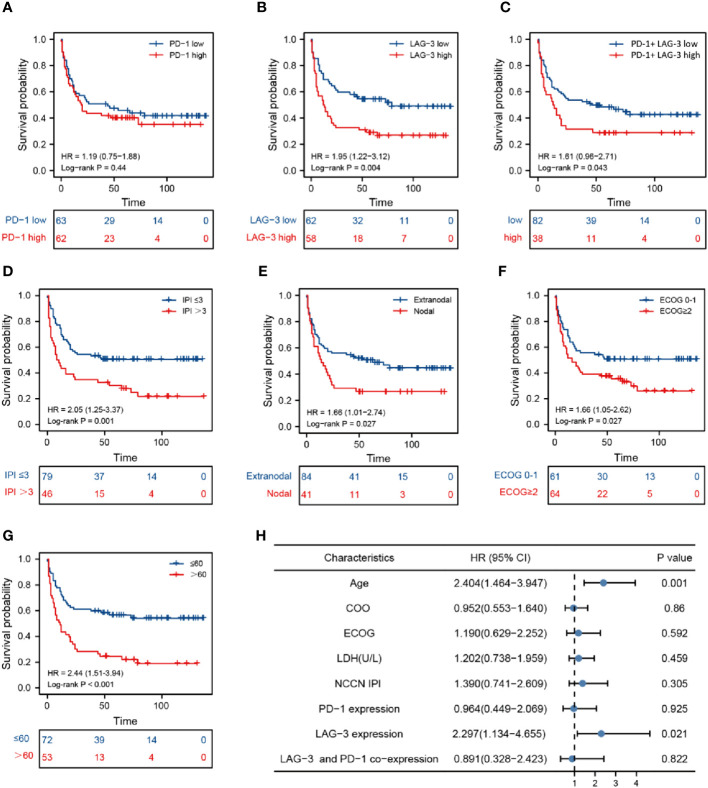
Prognosis analysis based on marker levels in DLBCL tissues. Kaplan-Meier analysis of patients with high and low PD-1 levels **(A)**, high and low LAG-3 levels **(B)**, high and low PD-1 and LAG-3 co-expression **(C)**, National Comprehensive Cancer Network-International Prognostic Index ≤ 3 and > 3 **(D)**, nodal and extranodal **(E)**, Eastern Cooperative Oncology Group score 0-1 and > 2 **(F)**, age > 60 and ≤ 60 years **(G)**. **(H)** Cox multivariate analysis of clinical prognosis of patients with DLBCL.

With the median expression levels of the PDCD1 and LAG-3 genes obtained from the database as the dividing line, the data of each group were divided into high expression group and low expression group in order to conduct subsequent analysis. The data of co-expression of PD-1 and LAG-3 was selected from the samples that overlap in expression between PD-1 and LAG-3 samples. There was no significant difference in overall survival (OS) between patients with DLBCL classified into two groups (high and low) according to PD-1 expression (**Figure 3A**), whereas OS was lower in patients with DLBCL in the high LAG-3 expression group than in those with low LAG-3 expression (**Figure 3B**). Furthermore, patients with elevated levels of both LAG-3 and PD-1 were characterized by shorter survival time and inferior prognosis (**Figure 3C**). Survival time of patients in National Comprehensive Cancer Network-International Prognostic Index (NCCN-IPI) group ≤ 3 was longer, and patients had better prognosis (*P* = 0.001) (**Figure 3D**), while the survival time of patients in the nodal, ECOG ≥ 2, and older age groups was shorter, indicating worse prognosis (**Figures 3E–G**). Non-germinal central B cell-like (GCB) phenotype, abnormal LDH or β2-microglobulin (β2-MG) levels, sex, B symptoms (fever, weight loss, and night sweats), Ann-Arbor stage, and site of invasion were not significantly associated with DLBCL disease prognosis (**Supplementary Figures 1A–G**). Multivariate Cox regression indicated that LAG-3 levels and age were independent risk factors associated with DLBCL (**Figure 3H**).

### 3.3 LAG-3 and PD-1 levels were positively correlated with infiltrating immune cells in DLBCL

**Figure 4:**
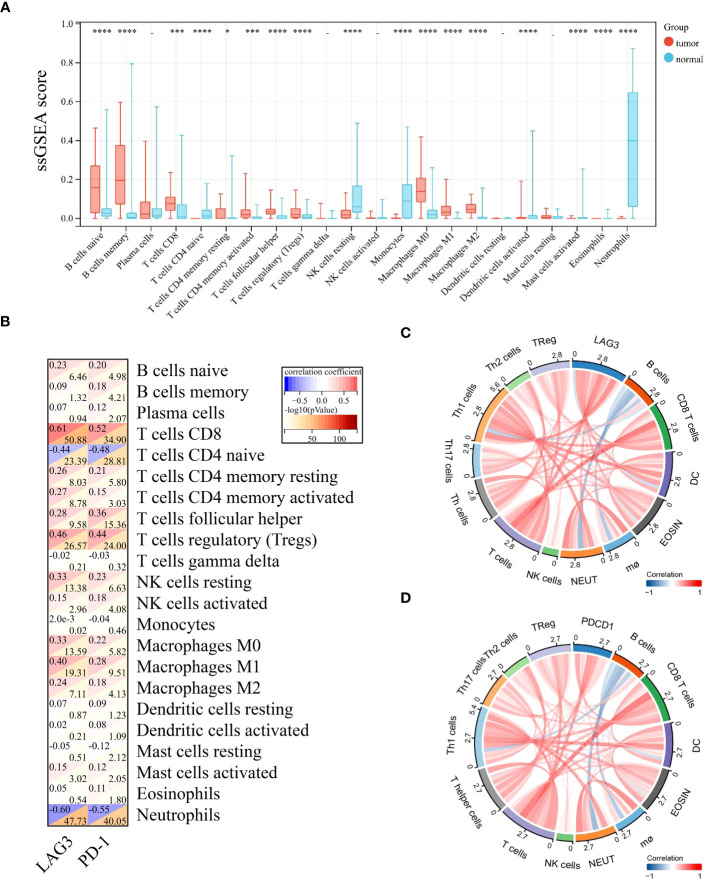
LAG-3 and PD-1 levels in diffuse large B-cell lymphoma were positively correlated with most infiltrating immune cells. **(A)** Differences in levels of 22 TILs in tumor and normal tissues. **(B)** Correlation of LAG-3 and PD-1 levels with those of 22 TILs in tumors. **(C)** Chord diagram of the results of correlation analysis between LAG-3 and TILs. **(D)** Chord diagram of the results of correlation analysis between PD-1 and TILs. Significance was defined as *P<0.05, ***P<0.001 and ****P<0.0001.

To better understand the differential immunological function of DLBCL and normal tissue, we used CIBERSORT and ssGSEA. The analysis revealed that DLBCL tissues had markedly elevated immune cell fractions (**Figure 4A**, **Supplementary Figure 2**). Additionally, LAG-3 and PD-1 expression levels were closely associated with most TILs (**Figure 4B**). LAG-3 levels were strongly positively correlated with T cells, Th1 cells, dendritic cells (DCs), CD8 T cells, eosinophils, T helper cells, neutrophils, Th17 cells, and Th2 cells, and strongly negatively correlated with B cells (**Figure 4C**). No correlations were detected between PD-1 levels and Th17 cells, Tregs, DCs, B cells, macrophages, neutrophils, or natural killer cells (**Figure 4D**).

### 3.4 Over-expression of LAG-3 and PD-1 on peripheral blood CD8^+^ T Cells from patients with DLBCL

**Figure 5:**
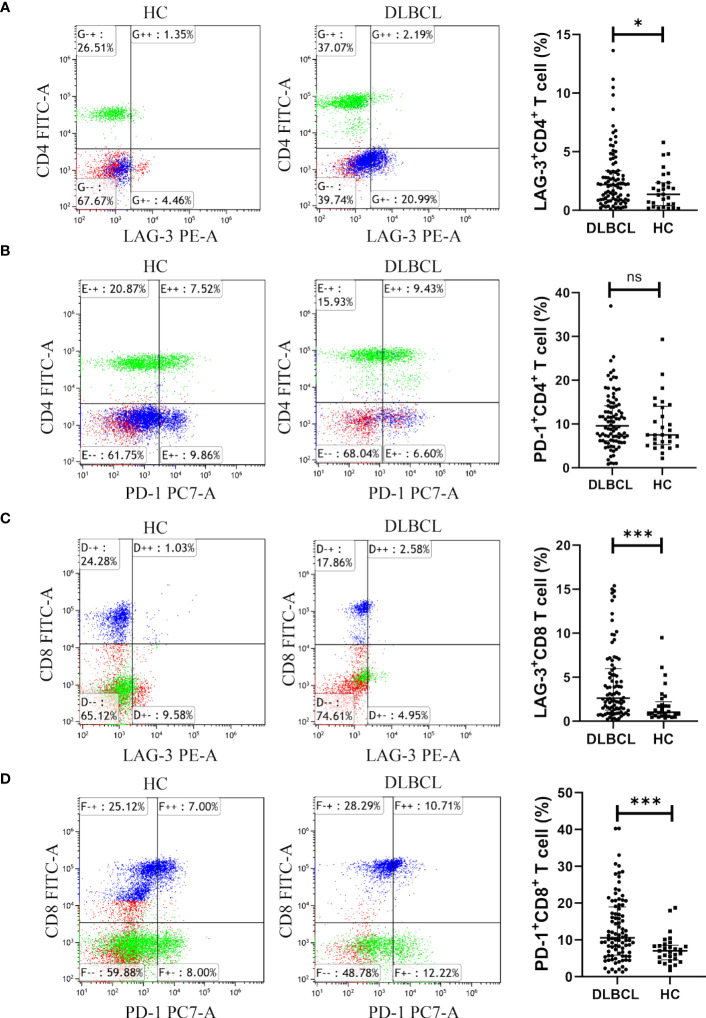
Expression of LAG-3 and PD-1 in CD4^+^ or CD8^+^ T cells in peripheral blood from the DLBCL and control groups. **(A, B)** Typical flow images of LAG-3^+^CD4^+^ T and PD-1^+^CD4^+^ T cells from patients with DLBCL and control groups and comparison plot. **(C, D)** Typical flow images of LAG-3^+^CD8^+^ T and PD-1^+^CD8^+^ T cells from patients with DLBCL and control groups and comparison plot. Significance was defined as *P<0.05, ***P<0.001. ns was defined as 'Not Significant'.

Levels of CD8^+^ T cells and CD4^+^CD8^+^ T cells in the peripheral blood of patients with DLBCL were higher than those in the healthy control (HC) group, while CD4^+^ T cell and CD4^+^/CD8^+^ T cell levels were lower than those in the HC group (**Supplementary Figures 3A–D**). LAG-3 expression on CD4^+^ T cells was higher in the peripheral blood of patients with DLBCL than those in HC samples (**Figure 5A**). There was no significant difference in PD-1^+^CD4^+^ T cells between the two groups (**Figure 5B**). In patients with DLBCL, levels of LAG-3^+^CD8^+^ T cells and PD-1^+^CD8^+^ T cells were significantly higher than those in HC (**Figures 5C, D**).

**Figure 6:**
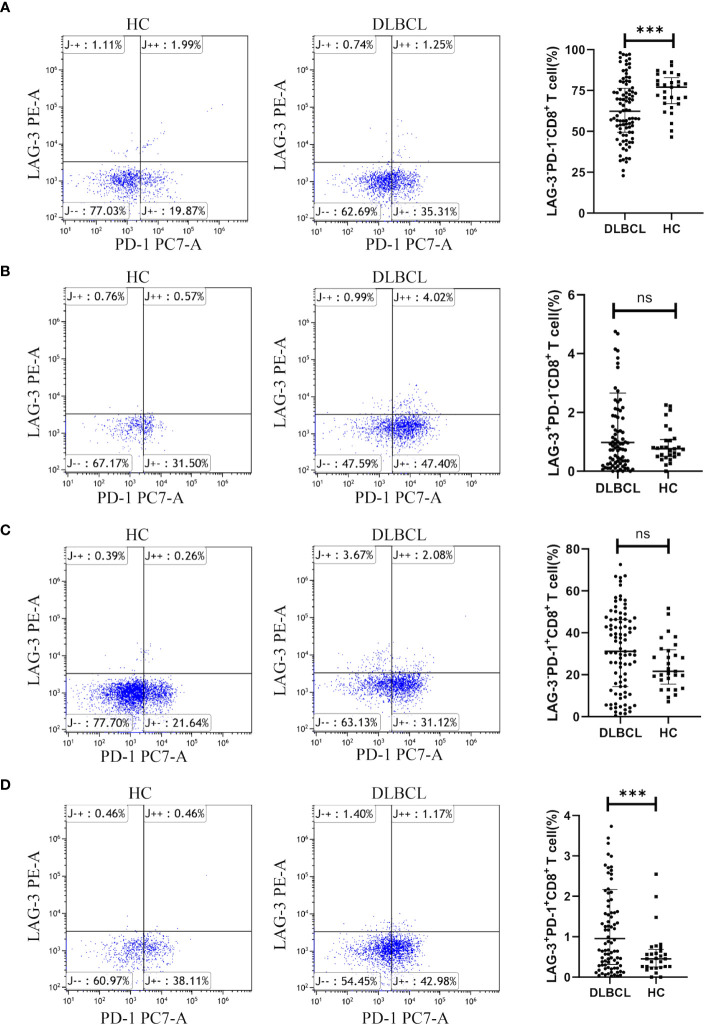
Expression of LAG-3 and PD-1 on CD8^+^ T cells in peripheral blood from patients with DLBCL and healthy controls. Typical flow images of LAG-3^-^PD-1^-^CD8^+^ T cells **(A)**, LAG-3^+^PD-1^-^CD8^+^ T cells **(B)**, LAG-3^-^PD-1^+^CD8^+^ T cells **(C)**, and LAG-3^+^PD-1^+^CD8^+^ T cells **(D)** from patients with DLBCL and control groups and corresponding comparison plot. Significance was defined as ***p<0.001. ns, no significance.

Additionally, patients with DLBCL had significantly lower levels of peripheral blood LAG-3^-^PD-1^-^CD8^+^ T cells (**Figure 6A**), while those of LAG-3^+^PD-1^+^CD8^+^ T cells were significantly higher in DLBCL than in HC (**Figure 6D**). There was no significant difference between the two groups in LAG-3^+^PD-1^-^CD8^+^ T or LAG-3^-^PD-1^+^CD8^+^ T cells (**Figures 6B, C**).

### 3.5 The impact of LAG-3 and PD-1 on CD8^+^ T cell function

**Figure 7:**
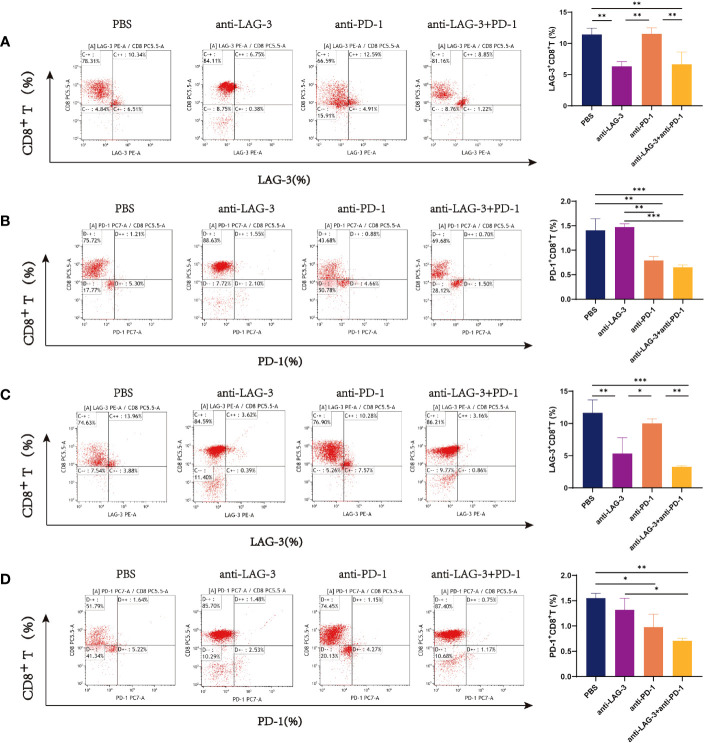
Flow cytometry images of LAG-3 and PD-1 on CD8^+^ T cells co-cultured with CD8^+^ T and SU-DHL6/OCI-LY3 cells. **(A)** Flow pattern of LAG-3 expression of CD8^+^ T cells after co-culture with CD8^+^ T and SU-DHL6 cells and corresponding comparison plot. **(B)** Flow patterns of PD-1 expression of CD8^+^ T cells co-cultured with SU-DHL6 after different treatments and corresponding comparison plot. **(C)** Flow patterns of LAG-3 expression of CD8^+^ T cells after co-culture with OCI-LY3 cells and different treatments and corresponding comparison plot. **(D)** Typical flow diagram of PD-1 expression on CD8^+^ T cells after co-culture with OCI-LY3 cells and corresponding comparison plot. Significance was defined as *P<0.05, **P<0.01, ***P<0.001.

CD8^+^ T cells from patients with DLBCL were purified (purity > 90%) using magnetic beads (**Supplementary Figure 4**). Then, we examined the expression of LAG-3 and PD-1 on CD8^+^ T cells after co-culture with SU-DHL6 or OCI-LY3 cells and treatment with anti-LAG-3 and (or) anti-PD-1 blocking agents. After administration of anti-LAG-3, LAG-3 expression was significantly decreased without affecting PD-1 expression. Similarly, anti-PD-1 treatment decreased PD-1 expression without affecting LAG-3 expression (**Figures 7A–D**).

**Figure 8:**
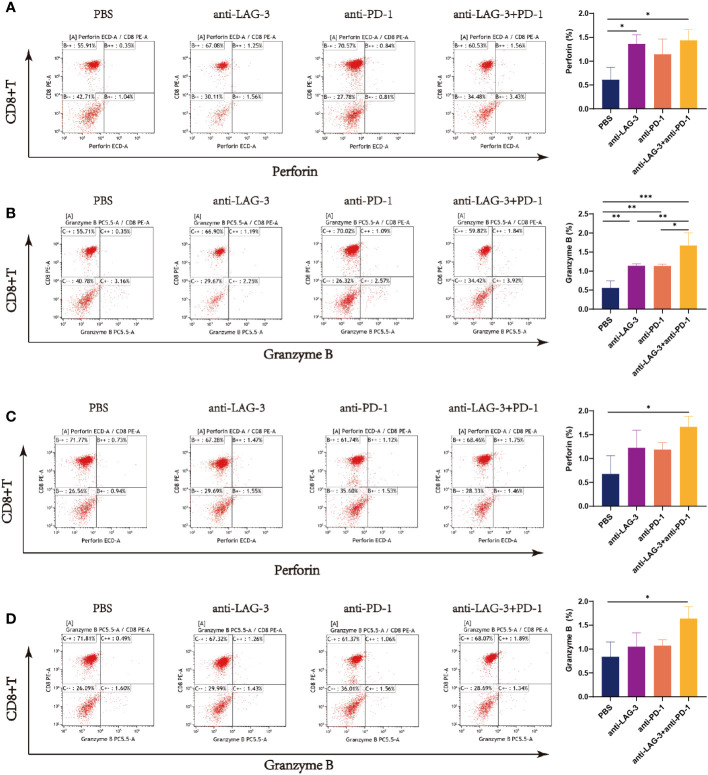
Flow cytometry analysis of perforin and granzyme B secretion by CD8^+^ T cells in the SU-DHL6/OCI-LY3 co-culture group. Typical flow images of perforin expression are shown for each group of CD8^+^ T cells co-cultured with SU-DHL6 **(A)** or OCI-LY3 **(C)** cells and corresponding comparison plot. Typical flow images of granzyme B expression are shown for each group of CD8^+^ T cells co-cultured with SU-DHL6 **(B)** or OCI-LY3 **(D)** cells and corresponding comparison plot. Significance was defined as *P<0.05, **P<0.01, ***P<0.001.

Levels of perforin were higher in CD8^+^ T cells from patients with DLBCL cultured with SU-DHL6 or OCI-LY3 cells after treatment with anti-LAG-3, anti-PD-1, or a combination of the two, relative to the PBS control group, and levels were highest after combination therapy (**Figures 8A, C**). Similarly, levels of granzyme B were higher in the groups treated with blocking agents than in the untreated PBS group (**Figures 8B, D**).

### 3.6 Influence of LAG-3 and PD-1 on DLBCL tumor cell apoptosis

**Figure 9:**
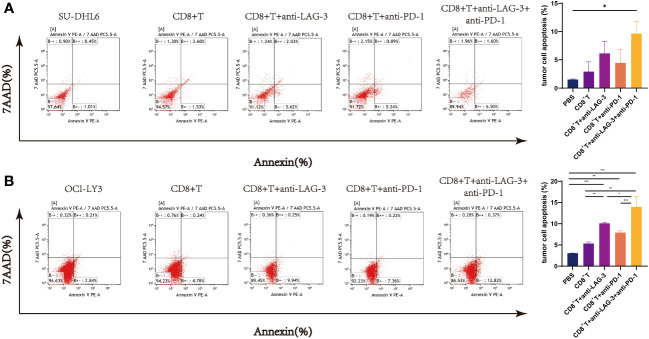
Flow cytometry analysis of SU-DHL6/OCI-LY3 tumor cell apoptosis. **(A)** Typical flow images of SU-DHL6 cell apoptosis in each group after different treatments of co-cultures of CD8^+^ T and SU-DHL6 cells and corresponding comparison plot. **(B)** Typical flow images of OCI-LY3 cell apoptosis in each group after different treatments of co-cultures of CD8^+^ T and OCI-LY3 cells and corresponding comparison plot. Significance was defined as *P<0.05, **P<0.01, ***P<0.001.

CD8^+^ T cells from patients with DLBCL were co-cultured with SU-DHL6 or OCI-LY3 cells and treated with LAG-3 and/or PD-1 blocking agents, followed by examination of SU-DHL6 or OCI-LY3 cell apoptosis. Administration of anti-LAG-3 or anti-PD-1 inhibitors, or a combination of both blocking agents, all induced a significant increase in overall SU-DHL6 and OCI-LY3 cell apoptosis rates (**Figures 9A, B**). These results indicate that increased LAG-3 and PD-1 on the surface of CD8^+^ T cells in patients with DLBCL result in impaired CD8^+^ T cells functionality, leading to their inability to kill tumor cells.

## 4 Discussion

DLBCL is intermediately to highly aggressive, grows rapidly, and is very heterogeneous, in terms of clinical manifestations, morphological characteristics, immunophenotype, genetic characteristics, treatment efficacy, and long-term prognosis, although the WHO has treated it as an independent disease (27). The application of monoclonal antibodies and immune targeted therapies have gained widespread interest and achieved certain curative effects; however, DLBCL remains an incurable disease, unless hematopoietic stem cell transplantation can be performed (28). Hence, there is an urgent need for new treatment approaches to overcome current limitations and enhance both patient survival time and quality of life. In this study, we first found that expression levels of LAG-3 and PD-1 were increased in patients with DLBCL. LAG-3 is considered a potential next-generation therapeutic target for human immunotherapy (29). According to related research reports (30), remarkable clinical efficacy can be achieved using PD-1 inhibitors to treat Hodgkin’s lymphoma and other aggressive B-cell lymphomas (31, 32). Treatment of DLBCL using PD-1 inhibitors is also of increasing general interest (33, 34); however, anti-PD-1 treatment has poor efficacy, with a less than 40% objective remission rate for the treatment of patients with relapsed/refractory DLBCL (35). We further clarified the expression and association with prognosis of LAG-3 and PD-1 in patients with DLBCL in this study.

The significant roles of both CD4^+^ and CD8^+^ T cells in the immune process of tumors in the body are widely recognized. However, the research findings indicated that there was no significant difference in the surface expression of PD-1 in CD4^+^ T cells between DLBCL patients and normal individuals in peripheral blood (*P* > 0.05), while there was a difference in LAG-3 expression (*P* < 0.05). In contrast, the expression differences of PD-1 and LAG-3 on the surface of CD8^+^ T cells in peripheral blood between DLBCL patients and normal individuals were highly significant (*P* < 0.001). The cytotoxic effects of CD8^+^ T cells on tumor cells are more direct, therefore we paid more attention to the influence of PD-1 and LAG-3 on the function of CD8^+^ T cells in the following experiment.

In this study, analysis of tissues by IHC and related clinical data from 137 cases of DLBCL demonstrated that LAG-3 and PD-1 were expressed at high levels and that their expression levels were positively correlated with one another. Multivariate Cox analysis suggested that high levels of LAG-3^+^ TILs may be an independent prognostic risk factor in patients with DLBCL. This experiment further confirmed that patients with DLBCL expressing high levels of LAG-3 have shorter OS, indicating that it can be a prognostic factor. Our data indicate that PD-1 therapy blocks only PD-1, and that PD-1 inhibitors will have little effect on T cells expressing high levels of LAG-3, which may lead to T cell function exhaustion. It has been proven in mouse cancer models that simultaneous blocking of LAG-3 and PD-1 co-expressed on CD4^+^ and CD8^+^ TILs can synergistically improve anti-tumor CD8^+^ T cell responses. We hypothesize that blockage of LAG-3 and PD-1 may improve PD-1 resistance for patients in whom PD-1 treatment is ineffective and tumors are drug-resistant; hence, LAG-3 and PD-1 blockage provides a potential new treatment approach for DLBCL.

LAG-3 expression reaches its highest point within 48 h of early T cell activation. If there is persistent viral infection or stimulation from tumor antigens, LAG-3 is expressed simultaneously with other immune checkpoints on the surfaces of both CD4^+^ and CD8^+^ T cells, indicating possible T cell dysfunction (36). Indeed, co-expression of LAG-3 and PD-1 in peripheral blood T cells from patients with lung cancer are reported to be biomarkers for T cell dysfunction (37). Zhao et al. observed up-regulated PD-1 expression in peripheral blood from patients with kidney cancer (38). Hence, LAG-3 and PD-1 may serve as biomarkers for assessing disease severity and predicting prognosis. Our results show that LAG-3 and PD-1 may be involved in the changes of CD4^+^/CD8^+^ cell balance in patients with DLBCL, which may be caused by down-regulation of the T cell immune response, influencing T cell immune response balance, and inhibiting immune responses, thereby inducing tumor cell immune evasion.

Abnormal expression of immune checkpoints may be related to disease progression and drug resistance. By blocking immune checkpoints, immunotherapy for malignant tumors aims to reactivate effector T cells to restore anti-tumor immune function, thereby allowing cytotoxic T cells to attack tumor cells and overcoming drug resistance to improve patient prognosis. Understanding the function of PD-1 and LAG-3 can in learning the basis for immunotherapy resistance. Encouraging results have been achieved by co-blocking LAG-3 and PD-1; for example, recent reports from the RELATIVITY-047 Phase III clinical trial indicated that the median progression-free survival time of the group with melanoma treated using relatlimab and nivolumab was 10.1 months, while that of the group receiving therapy targeting PD-1 alone was 4.6 months (39). In our study, after co-culture of CD8^+^ T cells from patients with DLBCL and SU-DHL6/OCI-LY3 cells *in vitro*, addition of LAG-3 and/or PD-1 inhibitors alone partially restored the function of CD8^+^ T cells, resulting in increased perforin and granzyme B secretion and an elevated proportion of tumor cells undergoing apoptosis. Taken together, our data demonstrate a synergistic effect between the separate immune targets, LAG-3 and PD-1, together, and show that higher LAG-3 and PD-1 expression in patients with DLBCL has strong inhibitory effects on CD8^+^ T cells. In addition, LAG-3 and/or PD-1 inhibitors can help restore immune cell function and provide a clinical approach for combined cellular immunotherapy of DLBCL.

## 5 Conclusion

In summary, our findings illustrate the crucial roles of LAG-3 and PD-1 in DLBCL. Further research on immunotherapy will be an important task over the next few years, and the influence of immunotherapy on immune checkpoint expression warrants comprehensive investigation. Immune targeted therapies diversify available treatment approaches, by providing us with new therapeutic methods, and bring new hope for elimination of malignant hematological tumors. Targeted immunotherapy of the LAG-3 and PD-1 pathways combined has great potential for clinical treatment of DLBCL.

## Data availability statement

The original contributions presented in the study are included in the article/**Supplementary Material**. Further inquiries can be directed to the corresponding author.

## Ethics statement

The study was approved by Institutional Review Board of Xinjiang Medical University Affiliated Tumor Hospital (No. K202011-1). The studies were conducted in accordance with the local legislation and institutional requirements. The participants provided their written informed consent to participate in this study. Written informed consent was obtained from the individual(s) for the publication of any potentially identifiable images or data included in this article.

## Author contributions

XL: Conceptualization, Formal Analysis, Funding acquisition, Methodology, Project administration, Resources, Supervision, Writing – review & editing. JM: Data curation, Formal Analysis, Software, Validation, Visualization, Writing – original draft. SY: Software, Validation, Writing – original draft. YZ: Validation, Writing – original draft. HY: Software, Validation, Writing – original draft. QZ: Methodology, Software, Writing – original draft.
