# Supplementary material for: Blockade of PD-1 and LAG-3 expression on CD8+ T cells promotes the tumoricidal effects of CD8+ T cells
Source: Front Immunol. 2023 Sep 28;14:1265255. doi: 10.3389/fimmu.2023.1265255 (PMC10568325; doi:10.3389/fimmu.2023.1265255)
Supplement: Supplementary file 1 [file DataSheet_1.docx]

**Blockade of PD-1 and LAG-3 Expression on CD8^+^ T cells Promotes the Tumoricidal Effects of CD8^+^ T Cells**

Jiajia Ma^1^, Shufang Yan^2^, Ying Zhao^3^, Huifang Yan ^1^, Qian Zhang ^1^, Xinxia Li ^1^*

*^1^ Department of Pathology, Xinjiang Medical University Affiliated Tumor Hospital, No. 789 Suzhou Dongjie, Urumqi, Xinjiang P.R. China;*

*^2^* *Department of Critical Care, Medicine of Karamay Central Hospital, No. 67, Junggar Road, Karamay city, Xinjiang P.R. China;*

*^3^* *The General Practice Department of the Third People’s Hospital of Xinjiang Uygur Autonomous Region, No. 9 Nanchang Road, Urumuqi, Xinjiang P.R. China;*

*** Correspondence:**

Xinxia Li, E-mail: lxxpatho@163.com; *Department of Pathology, Xinjiang Medical University Affiliated Tumor Hospital, No. 789 Suzhou Dongjie, Urumqi, Xinjiang, 830000, P.R. China;*

Supplementary Material legends

**Supplementary Figure 1. Prognosis analysis in DLBCL tissues.**

**Supplementary Figure 2. Twenty-two tumor-infiltrating immune cells in DLBCL and normal were estimated using the CIBERSORT algorithm.**

**Supplementary Figure 3. The T cell expression in peripheral blood of DLBCL and HC. Supplementary Figure 4. The CD8^+^ T cells were selected by magnetic beads. A. Magnetic bead sorting of DLBCL patients with lymphocyte gate. B. CD8^+^ T cell phylum in DLBCL patients.**

**Supplementary Table 1. Analysis of clinical characteristics of 137 cases of DLBCL.**

**Supplementary Table 2. Analysis of clinical data of peripheral blood in 100 cases of DLBCL.**

**Supplementary Table 3. Relationship between PD-1 and LAG-3 expression and clinicopathological parameters in DLBCL.**

**Supplementary Table 4. Relationship between PD-1 and LAG-3 co-expression and clinicopathological parameters in DLBCL.**

**Supplementary Figure 1. Prognosis analysis in DLBCL tissues.** Kaplan-Meier analysis of LDH < 250 and LDH≥250 (A), β2-Mg < 2.2 and β 2-Mg ≥2.2 (B), Male and Female (C), GCB and Non-GCB (D), Ann-Arbor I, II stage and III, IV Stage (E), other sites and PCNSL-DLBCL (F), with and without Symptom B (G).


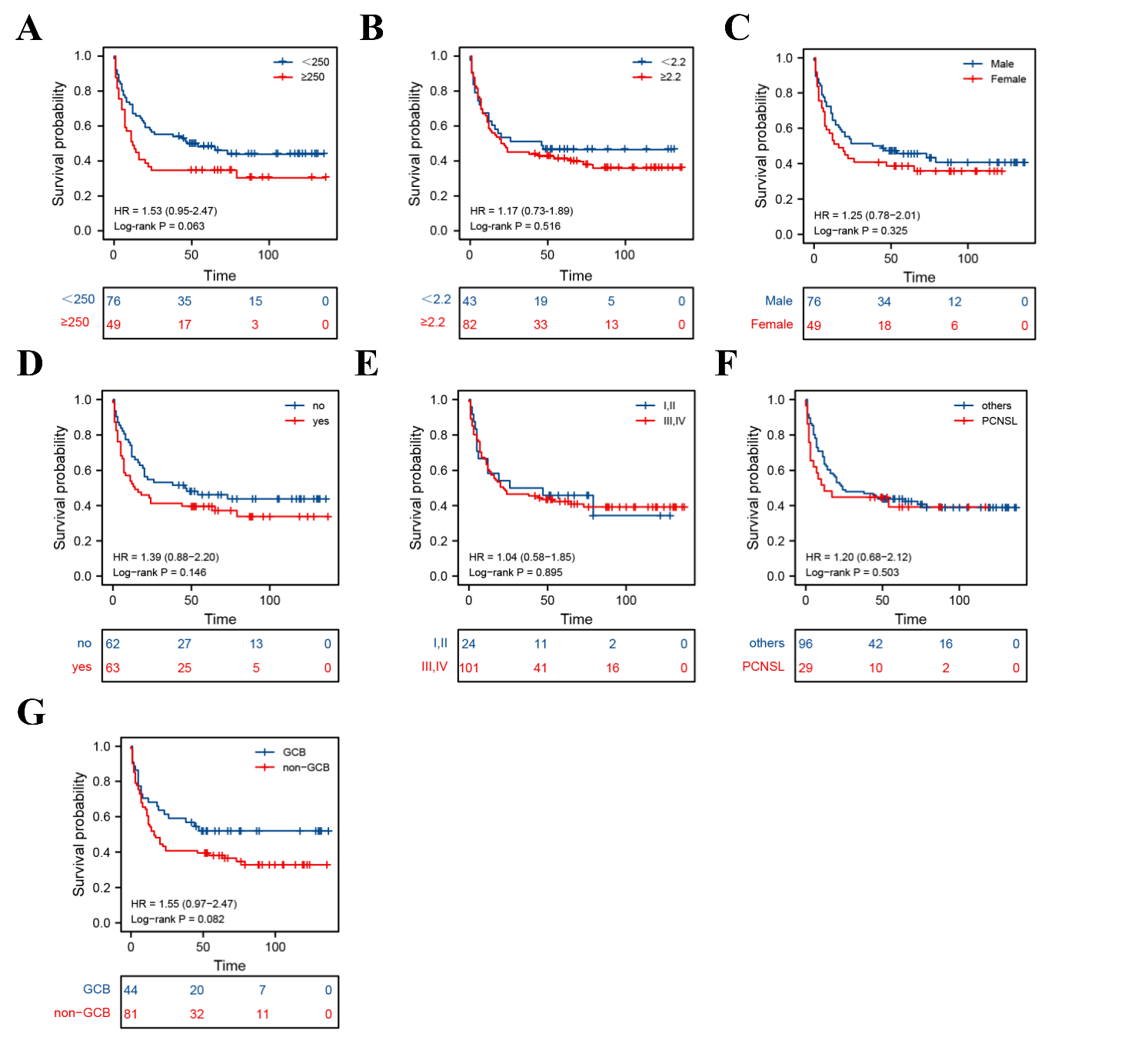


**Supplementary Figure 2. Twenty-two tumor-infiltrating immune cells in DLBCL and normal were estimated using the CIBERSORT algorithm.**


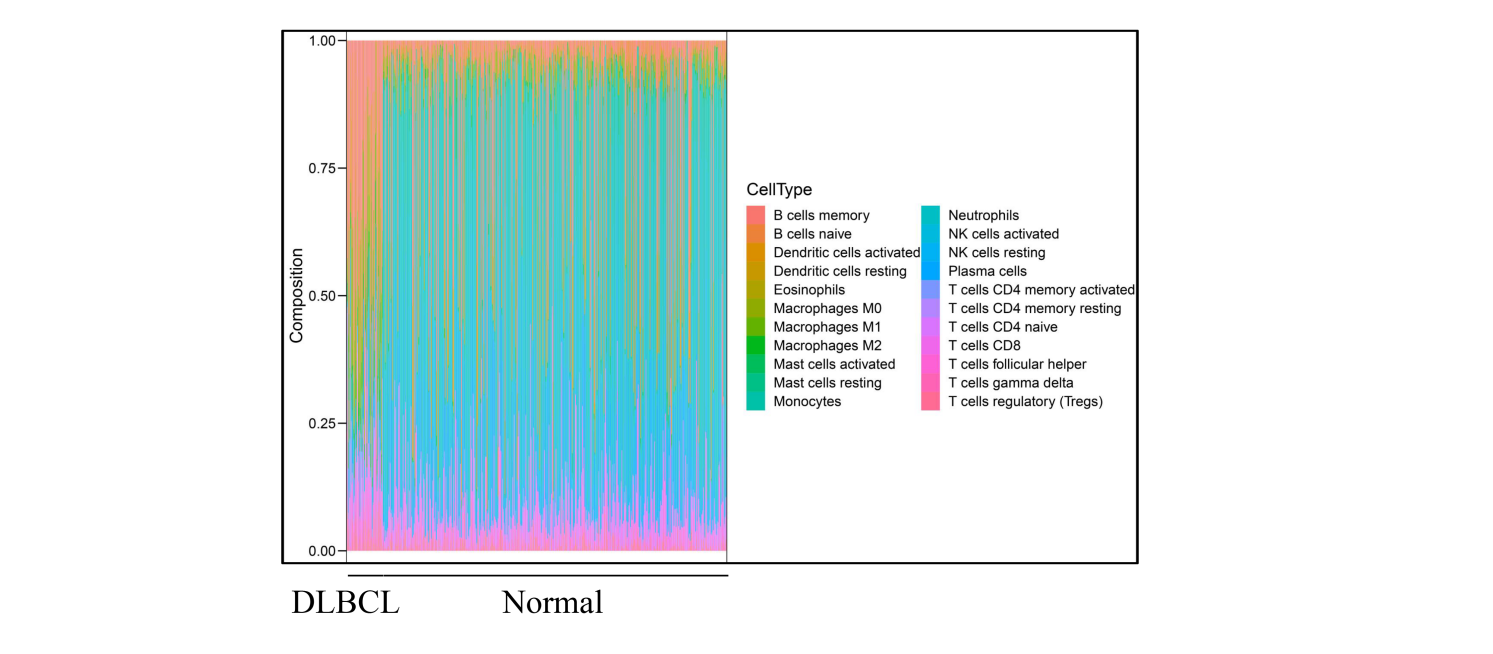


**Supplementary Figure 3. The T cell expression in peripheral blood of DLBCL and HC. A**. Comparison plot of DLBCL and HC in CD4^+^ T cells and typical flow image of CD4^+^ T in DLBCL patients (A1) and HC (A2). **B**. Comparison plot of DLBCL and HC in CD8^+^ T cells and typical flow image of CD8^+^ T in DLBCL patients (B1) and HC (B2). **C**. Comparison plot of DLBCL and HC in CD4^+^CD8^+^ T cells and typical flow image of CD4^+^CD8^+^ T in DLBCL patients (C1) and HC (C2). **D**. Comparison plot of DLBCL and HC in CD4^+^/CD8^+^ T cells.


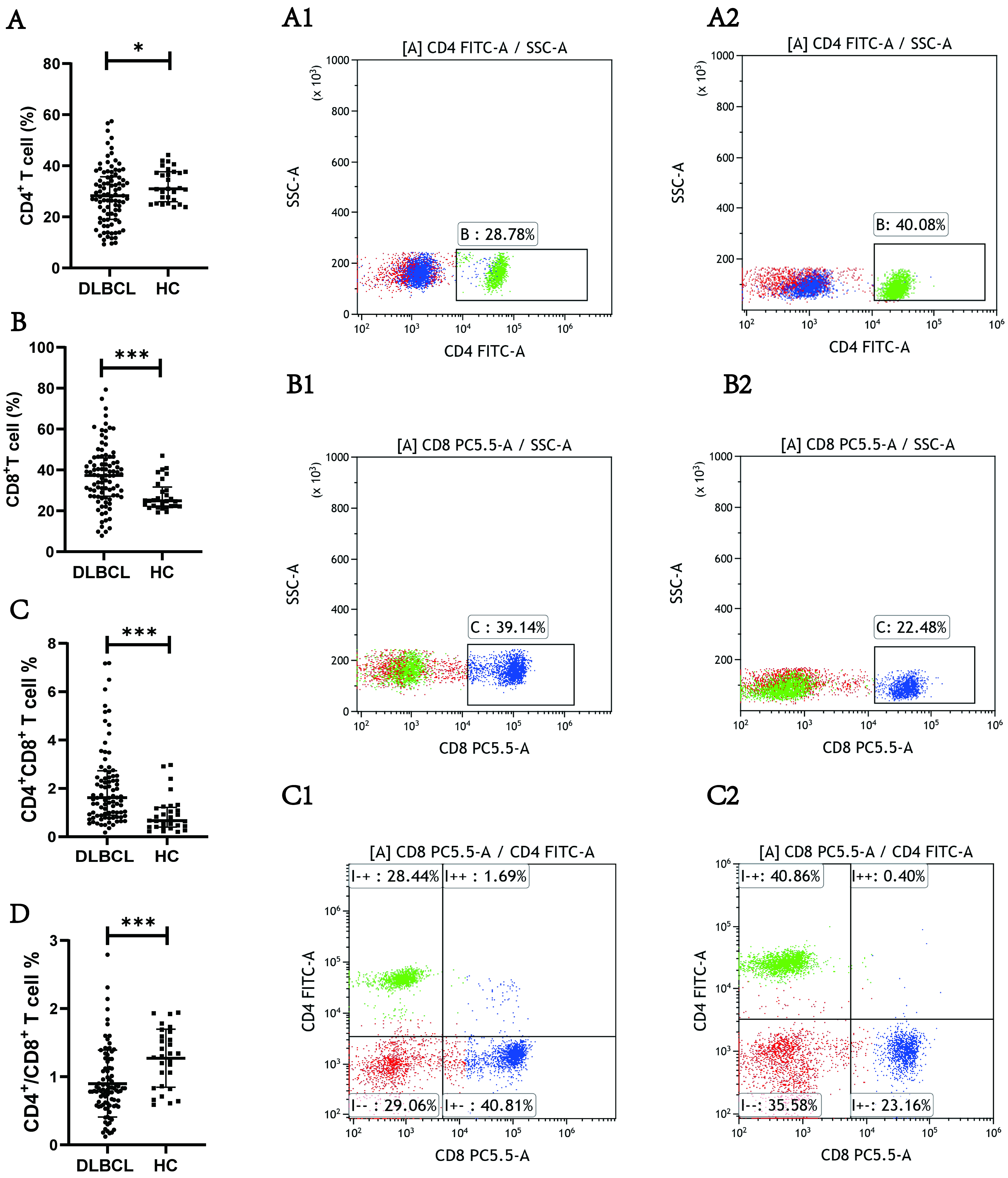


**Supplementary Figure 4. The CD8^+^ T cells were selected by magnetic beads. A**. Magnetic bead sorting of DLBCL patients with lymphocyte gate. **B**. CD8^+^ T cell phylum in DLBCL patients.

**
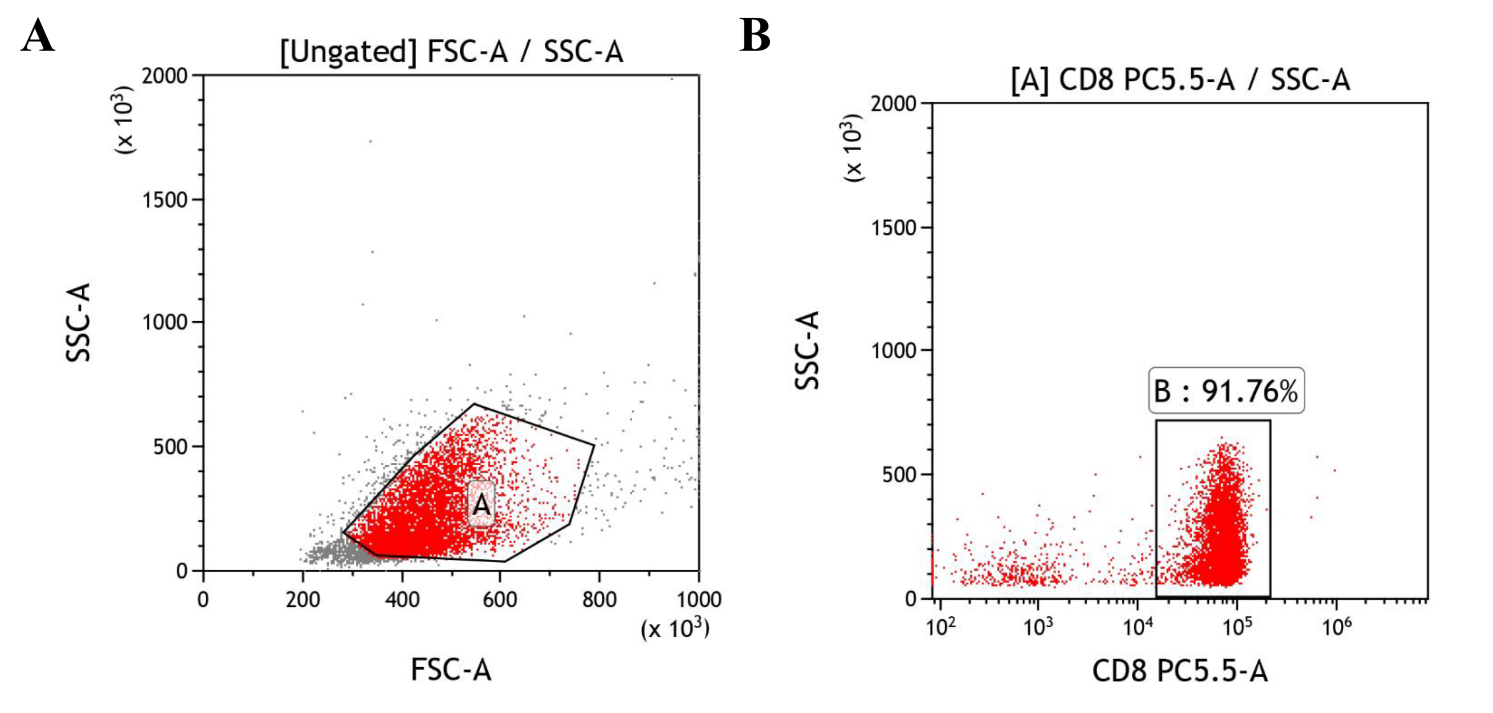
**

**Supplementary Table 1.** Analysis of clinical characteristics of 137 cases of DLBCL.

|  | | | | |
| --- | --- | --- | --- | --- |
| Clinical parameter | | N | | (%) |
| **Age** | |  | |  |
| ≤60 | | 76 | | 55.5 |
| ＞60 | | 61 | | 44.5 |
| **Gender** | |  | |  |
| Male | | 81 | | 59.1 |
| Female | | 56 | | 40.9 |
| **ECOG** | |  | |  |
| ECOG 0-1 | | 71 | | 51.8 |
| ECOG ≥2 | | 66 | | 48.2 |
| **Ann-Arbor stage** | |  | |  |
| I、II | | 27 | | 19.7 |
| III、IV | | 110 | | 80.3 |
| **Primary site** | |  | |  |
| Nodal | | 45 | | 32.8 |
| Extranodal | | 92 | | 67.2 |
| **LDH (U/L)** | |  | |  |
| ＜250 | | 84 | | 61.3 |
| ≥250 | | 53 | | 38.7 |
| **β2-MG (mg/L)** | |  | |  |
| ＜2.2 | | 48 | | 61.3 |
| ≥2.2 | | 89 | | 38.7 |
| **NCCN-IPI score** | |  | |  |
| ≤3 | | 86 | | 62.8 |
| ＞3 | | 51 | | 37.2 |
| **Subtype** | |  | |  |
| GCB | | 46 | | 33.6 |
| Non-GCB | | 91 | | 66.4 |
| **B** **symptom** | |  | |  |
| Yes | | 70 | | 51.1 |
| No | | 67 | | 48.9 |
| **PCNSL-DLBCL**  Yes | | 31 | | 22.6 |
| No | | 106 | | 77.4 |
|  |  | |  | |

**Supplementary Table 2.** Analysis of clinical data of peripheral blood in 100 cases of DLBCL.

|  | | |
| --- | --- | --- |
| Clinical parameter | N | (%) |
| **Age** |  |  |
| ≤60 | 53 | 53 |
| ＞60 | 47 | 47 |
| **Gender** |  |  |
| Male | 45 | 45 |
| Female | 55 | 55 |
| **ECOG** |  |  |
| ECOG 0-1 | 79 | 79 |
| ECOG ≥2 | 21 | 21 |
| **Ann-Arbor stage** |  |  |
| I、II | 38 | 38 |
| III、IV | 62 | 62 |
| **Primary site** |  |  |
| Nodal | 34 | 34 |
| Extranodal | 66 | 66 |
| **LDH (U/L)** |  |  |
| ≤250 | 56 | 57.1 |
| ＞250 | 42 | 42.9 |
| **β2-MG (mg/L)** |  |  |
| ≤2.2 | 32 | 32.7 |
| ＞2.2 | 66 | 67.3 |
| **IPI score** |  |  |
| ≤2 | 55 | 55 |
| ＞2 | 45 | 45 |
| **Subtype** |  |  |
| GCB | 26 | 27.1 |
| Non-GCB | 70 | 72.9 |
| **B symptom** |  |  |
| Yes | 21 | 21 |
| No | 79 | 79 |

**Supplementary Table 3.** Relationship between PD-1、LAG-3 expression and clinicopathological parameters in DLBCL.

|  | | | | | | | | | | |
| --- | --- | --- | --- | --- | --- | --- | --- | --- | --- | --- |
| Clinical parameter | N | PD-1 expression (N/%) | | χ^2^ | *P* | N | LAG-3 expression (N/%) | | χ^2^ | *P* |
|  |  | Low | High |  |  |  | Low | High |  |  |
| **Age** |  |  |  |  |  |  |  |  |  |  |
| ≤60 | 76 | 42(30.7) | 34(24.8) | 1.638 | 0.201 | 72 | 35(26.5) | 37(28.0) | 0.292 | 0.589 |
| >60 | 61 | 27(19.7) | 34(24.8) |  |  | 60 | 32(24.2) | 28(21.2) |  |  |
| **Gender** |  |  |  |  |  |  |  |  |  |  |
| Male | 81 | 44(32.1) | 37(27.0) | 1.241 | 0.265 | 76 | 41(31.1) | 35(26.5) | 0.729 | 0.393 |
| Female | 56 | 25(18.2) | 31(22.6) |  |  | 56 | 26(19.7) | 30(22.7) |  |  |
| **ECOG** |  |  |  |  |  |  |  |  |  |  |
| ECOG 0-1 | 71 | 43(31.4) | 28(20.4) | 6.132 | 0.013* | 68 | 35(26.5) | 33(25.0) | 0.029 | 0.866 |
| ECOG ≥2 | 66 | 26(19.0) | 40(29.2) |  |  | 64 | 32(24.2) | 32(24.2) |  |  |
| **Ann-Arbor stage** | |  |  |  |  |  |  |  |  |  |
| I、II | 27 | 9(6.6) | 18(13.1) | 3.902 | 0.048* | 26 | 10(7.6) | 16(12.1) | 1.958 | 0.162 |
| III、IV | 110 | 60(43.8) | 50(36.5) |  |  | 106 | 57(43.2) | 49(37.1) |  |  |
| **Primary site** |  |  |  |  |  |  |  |  |  |  |
| Nodal | 45 | 24(17.5) | 21(15.3) | 0.236 | 0.627 | 43 | 16(12.1) | 27(20.5) | 4.684 | 0.030* |
| Extranodal | 92 | 45(32.8) | 47(34.3) |  |  | 92 | 51(38.6) | 38(28.8) |  |  |
| **LDH(U/L)** |  |  |  |  |  |  |  |  |  |  |
| ＜250 | 84 | 41(29.9) | 43(31.4) | 0.210 | 0.647 | 81 | 47(35.6) | 34(25.8) | 4.430 | 0.035* |
| ≥250 | 53 | 28(20.4) | 25(18.2) |  |  | 51 | 20(15.2) | 31(23.5) |  |  |
| **β2-MG (mg/L)** |  |  |  |  |  |  |  |  |  |  |
| ＜2.2 | 48 | 22(16.1) | 26(19.0) | 0.607 | 0.436 | 46 | 23(17.4) | 23(17.4) | 0.016 | 0.899 |
| ≥2.2 | 89 | 47(34.3) | 42(30.7) |  |  | 86 | 44(33.3) | 42(31.8) |  |  |
| **NCCN-IPI score** | |  |  |  |  |  |  |  |  |  |
| ≤3 | 86 | 46(33.6) | 40(29.8) | 0.902 | 0.342 | 82 | 39(29.5) | 43(32.6) | 0.885 | 0.347 |
| ＞3 | 51 | 23(16.8) | 28(20.4) |  |  | 50 | 28(21.2) | 22(16.7) |  |  |
| **Subtype** |  |  |  |  |  |  |  |  |  |  |
| GCB | 46 | 21(15.3) | 25(18.2) | 0.615 | 0.433 | 44 | 26(19.7) | 18(13.6) | 1.834 | 0.176 |
| Non-GCB | 91 | 48(35.0) | 43(31.4) |  |  | 88 | 41(31.1) | 47(35.6) |  |  |
| **B symptom** |  |  |  |  |  |  |  |  |  |  |
| Yes | 67 | 31(22.6) | 36(26.3) | 0.880 | 0.348 | 66 | 36(27.3) | 30(22.7) | 0.758 | 0.384 |
| No | 70 | 38(27.7) | 32(23.4) |  |  | 66 | 31(23.5) | 35(26.5) |  |  |
| **PCNSL-DLBCL** |  |  |  |  |  |  |  |  |  |  |
| Yes | 31 | 13(9.5) | 18(13.1) | 1.139 | 0.286 | 29 | 15(11.4) | 14(10.6) | 0.014 | 0.906 |
| No | 106 | 56(40.9) | 50(36.5) |  |  | 103 | 52(39.4) | 51(38.6) |  |  |

**Supplementary Table 4.** Relationship between PD-1、LAG-3 co-expression and clinicopathological parameters in DLBCL.

| Relationship between PD-1、LAG-3 co-expression and clinicopathological parameters in DLBCL | | | | | | | |
| --- | --- | --- | --- | --- | --- | --- | --- |
| Clinical parameter | N | PD-1、LAG-3 co-expression (N/%) | | | | χ^2^ | *P* |
|  |  | Low | High | | |  |  |
| **Age** |  |  |  | | |  |  |
| ≤60 | 72 | 50(37.9) | 22(16.7) | | 0.881 | | 0.348 |
| >60 | 60 | 37(28.0) | 23(17.4) | | |  |  |
| **Gender** |  |  |  |  | | |  |
| Male | 76 | 53(40.2) | 23(17.4) | | | 1.168 | 0.280 |
| Female | 56 | 34(25.8) | 22(16.7) | | |  |  |
| **ECOG** |  |  |  | | |  |  |
| ECOG 0-1 | 68 | 46(34.8) | 22(16.7) | | | 0.189 | 0.604 |
| ECOG ≥2 | 64 | 41(31.1) | 23(17.4) | | |  |  |
| **Ann-Arbor stage** |  |  |  | | |  |  |
| I、II | 26 | 12(9.1) | 14(10.6) | | | 5.624 | 0.018* |
| III、IV | 106 | 75(56.8) | 31(23.5) | | |  |  |
| **Primary site** |  |  |  | | |  |  |
| Nodal | 43 | 26(19.7) | 17(12.9) | | | 0.841 | 0.359 |
| Extranodal | 89 | 61(46.2) | 28(21.2) | | |  |  |
| **LDH (U/L)** |  |  |  | | |  |  |
| ＜250 | 81 | 54(48.8) | 27(51.2) | | | 0.054 | 0.817 |
| ≥250 | 51 | 33(52.8) | 18(47.2) | | |  |  |
| **β2-MG (mg/L)** |  |  |  | | |  |  |
| ＜2.2 | 46 | 29(22) | 17(12.9) | | | 0.258 | 0.611 |
| ≥2.2 | 86 | 58(43.9) | 28(21.2) | | |  |  |
| **NCCN-IPI score** |  |  |  | | |  |  |
| ≤3 | 82 | 55(41.7) | 27(20.5) | | | 0.131 | 0.718 |
| ＞3 | 50 | 32(24.2) | 18(13.6) | | |  |  |
| **Subtype** |  |  |  | | |  |  |
| GCB | 44 | 31(23.5) | 13(9.8) | | | 0.607 | 0.436 |
| Non-GCB | 88 | 56(42.4) | 32(24.2) | | |  |  |
| **B symptom** |  |  |  | | |  |  |
| Yes | 66 | 44(33.3) | 22(16.7) | | | 0.034 | 0.854 |
| No | 66 | 43(32.6) | 23(17.4) | | |  |  |
| **PCNSL-DLBCL** |  |  |  | | |  |  |
| Yes | 29 | 19(14.4) | 10(7.6) | | | 0.003 | 0.960 |
| No | 103 | 68(51.5) | 35(26.5) | | |  |  |
